# Utilization of health services in a resource-limited rural area in Kenya: Prevalence and associated household-level factors

**DOI:** 10.1371/journal.pone.0172728

**Published:** 2017-02-27

**Authors:** Anthony K. Ngugi, Felix Agoi, Megan R. Mahoney, Amyn Lakhani, David Mang’ong’o, Esther Nderitu, Robert Armstrong, Sarah Macfarlane

**Affiliations:** 1 Centre for Population Health Sciences, Faculty of Health Sciences—East Africa, Aga Khan University, Nairobi, Kenya; 2 Department of Community Health, Faculty of Health Sciences—East Africa, Aga Khan University, Mombasa, Kenya; 3 Department of Medicine, Stanford University, Stanford, California, United States of America; 4 Department of Family Medicine, Faculty of Health Sciences—East Africa, Aga Khan University, Nairobi, Kenya; 5 Sub-County Health Management, Kaloleni Sub-County, Mariakani, Kilifi County, Kenya; 6 School of Nursing and Mid-wifery, Aga Khan University–East Africa, Nairobi, Kenya; 7 Medical College, Faculty of Health Sciences—East Africa, Aga Khan University, Nairobi, Kenya; 8 Global Health Sciences, University of California San Francisco, San Francisco, California, United States of America; Kenya Medical Research Institute—Wellcome Trust Research Programme, KENYA

## Abstract

**Background and methods:**

Knowledge of utilization of health services and associated factors is important in planning and delivery of interventions to improve health services coverage. We determined the prevalence and factors associated with health services utilization in a rural area of Kenya. Our findings inform the local health management in development of appropriately targeted interventions. We used a cluster sample survey design and interviewed household key informants on history of illness for household members and health services utilization in the preceding month. We estimated prevalence and performed random effects logistic regression to determine the influence of individual and household level factors on decisions to utilize health services.

**Results and conclusions:**

1230/6,440 (19.1%, 95% CI: 18.3%-20.2%) household members reported an illness. Of these, 76.7% (95% CI: 74.2%-79.0%) sought healthcare in a health facility. The majority (94%) of the respondents visited dispensary-level facilities and only 60.1% attended facilities within the study sub-counties. Of those that did not seek health services, 43% self-medicated by buying non-prescription drugs, 20% thought health services were too costly, and 10% indicated that the sickness was not serious enough to necessitate visiting a health facility. In the multivariate analyses, relationship to head of household was associated with utilization of health services. Relatives other than the nuclear family of the head of household were five times less likely to seek medical help (Odds Ratio 0.21 (95% CI: 0.05–0.87)). Dispensary level health facilities are the most commonly used by members of this community, and relations at the level of the household influence utilization of health services during an illness. These data enrich the perspective of the local health management to better plan the allocation of healthcare resources according to need and demand. The findings will also contribute in the development of community-level health coverage interventions that target the disadvantaged household groups.

## Introduction

Access to and utilization of health services are key to improvement of health outcomes in low and middle income countries (LMICs). In these countries, knowledge of access to and utilization of health services is important in planning for health resource allocation to different levels of the health system and monitoring the achievement of universal health coverage (UHC), which the World Health Organization (WHO) advocates as a means to ensuring equity in the use of health services [1,2]. Furthermore, knowledge of barriers to health service utilization among poor and marginalized populations is essential in informing the design of interventions aimed at increasing coverage of services [1].

Although the Kenyan Constitution enacted in 2010 declared health to be a universal right [2], progress towards UHC in Kenya has been limited [3,4]. There is also inadequate population-level information on utilization of health services. A recent healthcare benefit incidence study in Kenya showed inequitable distribution of services according to ability to pay rather than need for care [5].

In the context of recent devolution of health and development functions from central to county governments [6], information on access and utilization of services is critical to county health systems managers particularly in rural and marginalized areas with poorer population health indicators. In lieu of competition for scarce resources between health and other development functions at the local level, it is crucial to identify and describe key determinants of health e.g. access and utilization of services and factors that influence these determinants amongst the poorest and most vulnerable segments of the population. Knowledge of such determinants and associated factors is key in designing and delivery of finely targeted interventions that save on scarce local resources and promise accelerated improvement of health outcomes.

As part of a partnership with the local sub-county health management to strengthen primary healthcare, we determined the prevalence of health services utilization by residents reporting a health complaint in the month preceding the survey and its associated factors in one of the poorest rural populations in Kenya.

## Methods

We undertook a cross-sectional survey using the WHO cluster-sample design [7] because we did not have an adequate sampling frame or up-to-date vital statistics for the study area.

### Study area and population

We conducted this study in Kaloleni and Rabai sub-counties (formerly Kaloleni district) in the coastal area of Kenya. The two sub-counties cover an area of 909 km^2^ and have a population of about 290,000 living in about 44,000 households [8]. There are few indicator data for this area, and thus the figures usually presented are for the larger Kilifi county in which the two sub-counties are located. Kaloleni/Rabai sub-counties are among the poorest regions of Kenya [9]. This is an area of the Aga Khan University (East Africa) population-level programming and was selected in consultation with the County government due to lack of population-level health research and suspected poor population health indicators relative to the rest of Kilifi county. According to the 2009 Kenya National Population and Housing Census, children under five years of age comprise 20% of the Kilifi population and women of reproductive age comprise another 25%. Fifty seven percent of the population are Christian, 19% are Muslim and the remainder are traditionalists [8]. Approximately 70% of the population lives below the poverty line and 81% rely on subsistence agriculture, crafts and petty trading for their livelihoods. Maternal, neonatal and child health indicators are poorer than the national averages [10].

The study area has three administrative divisions (Rabai, Mariakani, and Kaloleni) which are sub-divided into a total of 12 locations and 34 sub-locations. Forty health facilities serve these sub-counties: 20 public/government health facilities (16 dispensaries, one health centre, one sub-district hospital, one district hospital, one military health centre), three faith-based facilities (one hospital and two dispensaries), three NGO dispensaries, and 14 privately owned dispensaries [11]. The doctor to population ratio (1 to 36,000) in this area is below the national average of 1 to 5,000 [12] and a nurse to population ratio of 1:1830.

### Sample size, sampling of households and data collection

We estimated that we required 30 clusters (specific villages) of 27 households each to meet the objectives of this survey. This sample size would be adequate to describe the demographic characteristics, illness episodes and utilization of health services of individuals in about 800 households. We picked the largest sample size possible, based on the assumption that each of the characteristics would have a 50% distribution in the study population and used a precision of 2.5% and a design effect of 2 to adjust for cluster sampling.

Using probabilities proportionate to the number of households in the sub-locations, we selected 23 of the 34 sub-locations from which we selected the thirty clusters. With the aid of local administration (chiefs or assistant chiefs), we listed all the villages in each of the selected sub-locations and then randomly selected the number of villages required in each. We enlisted the support of the local village elders to identify village boundaries and to select convenient starting points in each village (e.g. the village elder’s house, a community health worker’s house etc) and then sequentially selected 27 households to comprise the cluster. We piloted the study tool in a sub-location outside the selected study area (neighbouring Kwale County).

The enumerators administered the study questionnaire to consenting participants (the most senior male or female resident in the household) after obtaining written-informed consent from them. In the event the appropriate respondent was unavailable, they made an appointment to return at the earliest time convenient and followed-up for three consecutive times before replacement.

Besides obtaining the socio-demographic information, we collected data on history of sickness or health complaints for each member of the household in the preceding month, whether they sought healthcare services for the complaint, signs/symptoms of the condition, name of health facility visited, who refered them to the facility e.g. community health worker (CHW), self-referral or village elder among others, and reasons they did not seek services if they did not attend. We list the questions asked on history of illness and utilization of health services in S1 Appendix. Respondents reported a range of signs and symptoms of the index illness episode. Based on these, a family medicine physician (MRM) classified sick respondents into broad illness categories.

### Data management and analysis

Trained data entry clerks double entered and verified all data in Epi Info v7 [13]. We described the distribution of study subjects characteristics and compared the demographic parameters of those that were ill in the previous month and those who were not, and those who sought health services and those who did not among those that fell ill. We generated household socio-economic status (SES) quintiles based on reported ownership of a range of 15 household items and properties using principal component analyses (PCA) [14]. Bivariate associations between utilization of health facilities and potential predictors were examined by Pearson correlation. Covariates included: (i) relationship to the household head, (ii) age in years (grouped), (iii) sex, (iv) schooling (mother’s schooling for those < 18 years old), and (v) occupation of the household head, (vi) religion of household head, and (vii) the SES quintile for the household.

Multivariate analysis was conducted using a multilevel logistic mixed effect model with maximum likelihood (ML) estimation to determine the household-level factors associated with utilization of health services if a household member experienced an illness episode in the preceding month. We investigated the association between the utilization of health services and covariates (any factor with p<0.25 in the bivariate analyses). We used a four step model building procedure. As first step an unconditional model was tested with household and village as a random intercept to examine the variation in outcomes at these levels. An ICC was estimated to find the contribution of these levels on total variation. The household contributed 34% of the total variance in the outcome (S1 Table) and was therefore included as random effect in subsequent modelling while the village contributed 2.9% of the total variation and was thus excluded from further analyses. In the next step, relationship with head of household was added as a fixed effect and random slope, and likelihood ratio (LR) test was used to confirm whether the variance of the slope was significantly different from zero. Finally variables significant at p<0.25 in bivariate analysis were incorporated into model and retained if p<0.05. In the final model, we made adjustment for the interaction of age and relationship to the head household since these variables were correlated (p<0.001) in the bivariate analysis. All data cleaning and analyses were done in Stata v13 [15].

## Ethical approval

We obtained approval for the study from the Aga Khan University-East Africa Health Ethics Review Committee and the University of California San Francisco Committee on Human Research. All participants provided written informed consent. Since data were collected at the level of the household and only heads of household were interviewed, consent was obtained from household heads on behalf of all members whose information was sought. This approach was approved by both IRBs.

## Results

The enumerators collected data on 6,440 individuals from 829 respondents each representing a household between 14^th^ July and 29^th^ August 2014. Eighty five percent of the respondents were either the head of the household (20.2%) or their spouse (64.5%). Of the rest, 5.5% were the grown-up son/daughter of the household head and 5.4% were either his son or daughter-in-law. The median (IQR) age of the respondents was 47 (36–59) and 71.5% were male and 28.5% female.

A total of 624 (75.3%) of the households reported a sick member in the preceding month, with a median (IQR) of 1 (1–2) members per household. 1,230 (19.1%, 95% CI: 18.3–20.2) of the 6,440 household members had had a health complaint. Demographically, older residents (50+ years of age) and the heads of the household experienced higher frequencies of ill health compared to the rest of the population (Table 1). The distribution of study factors across the two categories of health service utilization among ill household members is shown in Table 1. Results of the bivariate correlation analyses showed significant correlation between age, relationship with head of household and utilization of health services during an illness (S2 Table).

**Table 1:** Distribution of sickness and utilization of health facilities by demographic characteristics.

		Sickness in the previous month		Utilization of HF visited when sick	
Characteristic		Fell ill n(row %)	Total	No n(row %)	Total
1) Relation to hhh^1^					
	Self	571 (69.2)	825	108 (19.0)	568
	Spouse	261 (40.9)	638	61 (23.5)	260
	Child	246 (8.7)	2,822	73 (30.2)	242
	Others	145 (6.8)	2,121	41 (28.9)	142
	Total	1,223 (19.1)	6,406	283 (23.4)	1,212
3) Age (years)					
	0–14	168 (5.3)	3,148	53 (32.3)	164
	15–25	170 (13.0)	1,303	52 (31.0)	168
	26–49	495 (40.5)	1,221	103 (20.9)	492
	50+	343 (52.4)	655	64 (18.7)	342
	Total	1,176 (18.6)	6,327	272 (23.3)	1,166
4) Sex					
	Male	600 (19.8)	3,024	131 (21.9)	597
	Female	624 (19.8)	3,416	152 (24.7)	616
	Total	1,224 (21.7)	6,440	283 (23.3)	1,213
5) Religion of hhh					
	Catholic	92 (19.1)	482	18 (19.6)	92
	Protestant	390 (21.0)	1,855	83 (21.6)	385
	Islam	483 (19.5)	2471	127 (26.7)	476
	Traditional	83 (17.6)	471	22 (26.5)	83
	None	148 (18.3)	807	22 (26.5)	142
	Other	8 (20.0)	40	4 (50.0)	8
	Total	1,204 (19.6)	6,126	281 (23.6)	1,191
6) Occupation of hhh					
	Bluecollar/technical	147 (21.7)	677	35 (24.0)	146
	Agriculture/crafts	648 (18.6)	3,477	147 (22.9)	641
	None	63 (18.4)	343	14 (22.2)	63
	Others	342 (21.2)	1613	85 (25.2)	337
	Total	1,200 (19.6)	6,110	281 (23.7)	1,187
7) SES quintile					
	1	251 (20.5)	1,222	63 (25.4)	248
	2	261 (18.7)	1,397	64 (24.6)	260
	3	246 (19.4)	1,266	46 (18.7)	246
	4	329 (20.3)	1,623	88 (27.3)	322
	5	105 (19.2)	547	18 (17.14)	105
	Total	1,192 (19.7)	6,055	279 (23.6)	1,181
8) Schooling					
	Yes			185 (22.1)	836
	No			82 (24.6)	334
	Total			267 (22.8)	1,170

^1^ Head of the household.

The most commonly reported illnesses/health conditions experienced by residents in the month preceding the survey included infectious (specific conditions e.g. malaria and those others associated with fever), respiratory (conditions associated with a cough, chest pain or flu), neurologic (conditions associated with a headache) and gastrointestinal (associated with stomach pain, diarrhoea or vomiting) (Table 2). 76.7% (95% CI: 74.2–79.0) of the 1,213 household members who respondents reported as having had a health complaint sought healthcare and of these, 76.4% self-referred or were taken to the health facility, mainly by parents or other relatives while the rest were referred by e.g. CHW, other health workers, teacher, local government administrator etc. Of the 23.3% that did not seek health services during their illness, 42.8% self-medicated by buying non-prescription drugs from either shops or pharmacies. Another 20% did not seek health care from facilities as it was too costly or they did not have money at the time. For 9.9% the sickness was not serious enough to necessitate visiting a health facility and for 8.1% the nearest health facility was too far or they did not have transport (Table 3).

**Table 2:** Health conditions experienced by residents in the month preceding the survey.

Disease conditions	Freq.	Percent
Infectious Disease	413	28.4
Respiratory	318	21.9
Neurologic	200	13.8
Gastrointestinal	195	13.4
Musculoskeletal	106	7.3
Dermatologic	65	3.0
Renal Problem	55	2.3
Cardiologic/Respiratory	34	1.5
Eye Problem	22	1.0
Cardiologic	14	3.0
Blood disorder	9	0.6
Dental	8	0.6
Ear Problem	6	0.4
Other	4	0.3
Reproductive Health	3	0.2
Drug Side effect	1	0.1
Psychiatric	1	0.1
**Total**	1,454*	**100**

*Some respondents were grouped into more than one category.

**Table 3:** Reasons given for failure to utilize health services.

Main reason given		Freq.	Percent
Cost related	Too costly/no finances	48	17.0
	Too far/no transport	23	8.1
Non-cost related	Facility not open/no staff	12	4.2
	Don't trust facility/poor services	5	1.8
	Not necessary/serious	19	6.7
	Not customary	2	0.7
	Visited traditional healer	5	1.8
Other reasons*:	** **		
Cost related	Too costly(2*)	14	4.9
	Self-medication	125	44.2
Non-cost related	Fear of drugs/injections	2	0.7
	Facility not open/no staff (2*)	2	0.7
	Not necessary/serious (2*)	9	3.2
	Patient too old	2	0.7
	No response	15	5.3
	**Total**	**283**	100.0

*Mentioned in the category “other” (where this was not the primary reason given).

The majority (96.9%) of respondents indicated that the nearest health facility was within the study area. The 913 residents who sought health care utilized services from 94 health facilities. However, among the 1,213 who fell sick, only 60.1% attended facilities that were within the study sub-counties and only 51.1% attended the identified health facility nearest to their home.

Nine out of ten of the most frequently utilized health facilities were located in the study area and were patronized by 53.0% of the respondents that sought health services, with an equal distribution between rural and urban locations. Eight out of ten facilities were public/government facilities and were mainly (8/10) in tier 2 (dispensary-level) of the health system. These most frequently utilized facilities were located in major towns of the study area (high population density) or were the only health facilities available within a wide radius in rural areas (Table 4). A large proportion (68.3%) of those who sought health services attended public health facilities, 30.9% visited privately owned facilities (including faith-based and private not-for-profit) while 4.3% (n = 5) of the facilities mentioned could not be found in the facilities master list [11], possibly because the respondents used unknown facility names. Overall, a majority (93.7%) of the sick respondents visited tier 2 (dispensary) health facilities.

**Table 4:** The top 10 most utilized health facilities in Kaloleni sub-county.

Health Facility	Freq. (%)	Type	Level	In study area?	Rural/Urban	Comment
Mariakani Hospital	101 (11.1)	Public	4	Yes	Urban	The main District hospital located in Mariakani town
Shangia dispensary	68 (7.4)	Public	2	Yes	Urban	Lower level facility in the main town of Mariakani
Kinarani dispensary	46 (5.0)	Public	2	Yes	Rural	Only dispensary in a 15km radius
Shika adabu dispensary	41 (4.5)	Public	2	No	Urban	In Mombasa (where most labour migrants reside)
Vishakani dispensary	41 (4.5)	Public	2	Yes	Rural	In the urban Kaloleni area
Gotani dispensary	40 (4.4)	Public	2	Yes	Rural	A model health centre (with 2 Clinical officers)
Kamkomani dispensary	40 (4.4)	Public	2	Yes	Rural	In the urban Mariakani area
Khadija clinic	38 (4.2)	Private	2	Yes	Urban	In Mazeras township
St. Luke’s hospital	36 (3.9)	Religious	4	Yes	Urban	Large level 4 hospital in the urban Kaloleni area
Kombeni dispensary	33 (3.9)	Public	2	Yes	Rural	The only public facility in a large area
Total	484					
Total as % of all sick	53.0					

We found that 34% of the total variation in the probability to utilize healthcare during an illness was attributable to random effects (unmeasured or unmeasurable confounders) operating at the level of the household or higher (S1 Table). In the multivariate analyses, relationship to head of household was the only factor associated with utilization of health services in the (p = 0.03) after adjusting for the interaction between age and relation to the household head. Relatives other than the immediate family of the head of household (including son/daughter in-law, grand child, parent/parent in-law, brother/sister, adopted child and other relatives) were 4.9 times less likely to seek medical help from a health facility when they fell ill (Table 5).

**Table 5:** Regression analyses factors associated with utilizing health facilities during illness.

Factor	Odds Ratio (95% CI)	P-value
1) Relation to hhh^1^		
Self	1.00	
Spouse	1.47 (0.54–4.00)	0.45
Child	na^†^	na
Other relatives	0.21 (0.05–0.87)	0.03
2) Age group (years)		
50+	1.00	
0–14	0.94 (0.04–23.4)	0.97
15–25	0.38 (0.08–1.84)	0.23
26–49	1.05 (0.55–1.98)	0.89
3) Relation to hhh*age group		
Spouse*0–14	0.13 (0.001–16.41	0.41
Spouse*15–25	0.65 (0.09–4.89)	0.68
Spouse*26–49	0.48 (0.14–1.60)	0.23
Child*0–14	na^†^	na
Child*15–25	na^†^	na
Child*26–49	na^†^	na
Others*0–14	1.49 (0.04–57.78	0.83
Others*15–25	4.37 (0.43–44.14)	0.21
Others*26–49	2.01 (0.34–11.92)	0.44

^1^ Head of the household

^†^ model did not converge as none (0) of those who failed to visit health facility when sick were 'child' of the hhh in the '50+' year age category.

## Discussion

We have estimated a prevalence of health services utilization that was similar to the 77.8% reported previously for Kenya [16]. We found that most of the respondents who fell ill but did not seek health services either self-medicated, felt that services were too costly (or did not have money to pay), or felt that the illness was not serious or that the facility was too far. Residents visited a surprisingly large number of health facilities outside of the study area although in terms of absolute number of patients, facilities within the study sub-counties accounted for majority of the visits. Tier 2 (Dispensary) facilities were the most commonly used. In the multivariate analyses, relatives who were not in the nuclear family of the head of the household were at significant disadvantage with regard to utilizing health services when they were ill. These included son/daughter in law, grandchild, parent/parent-in-law, brother/sister and adopted child among other relatives.

Conditions with an infectious aetiology (fevers, respiratory conditions, diarrhoea and dermatological conditions) were the most common causes of ill health in this population, accounting for 65% of health complaints in the previous month. This concurs with findings of a hospital-based study done in the same area in which community-acquired bacteremia was an important cause of paediatric morbidity and mortality [17]. Poverty-related determinants of infectious diseases such as malnutrition and under-nutrition [18], lack of access to safe drinking water and sanitation and inadequate availability of water for hygiene are pervasive in this poor rural community. Addressing these determinants will require a multisectoral approach between the county government, development partners and other stakeholders.

It was surprising that socio-economic status was not associated with utilization of health services as has been found elsewhere [19,20]. In our study, this was likely because our population was more or less homogeniously poorer than the rest of the country [9] and as such the socio-economic status determined using PCA may not clearly distinguish wealth groups in this population [14]. Indeed, a closer look at the data shows that our PCA generated SES scores that were skewed to the right (to the lower income gropus), ranging between -7.84 to 1.46 compared to a much more normally distributed range of -2.10 to 2.62 in the 2008–9 Kenya Demographic and Health Survey data [10]. Thus, it is possible that poverty related reasons were still partially responsible for non-utilization of health services.

The main reason for failure to utilize health services i.e. self medication is likely to be related to avoidance to pay user fees charged at the health facilities as well as costs associated with travel [21], since these respondents do not fall in the category that felt that the illness was not serious to warrant visiting a facility. The user fees charged, though minimal, could be significant for this impoverished population [22]. Taken together with those that actually indicated that seeking care was costly and transport to health facilities as the main reasons for not seeking healthcare, the costs associated with seeking care could account for appoximately 75% of the reasons for failure to do so. This is a rather high proportion, and an indication that distribution of health services (and benefits) even in the public sector could still be based on the ability to pay rather than the need for services, as was observed in a natiowide study [5].

Although the majority of those seeking healthcare utilized health services from health facilities within the study sub-counties, a significant proportion of health facilities patronized were from outside the study area or those not identified as the closest to the homestead. This being a largely poor rural area, it is unclear why residents would opt to seek health services far from home, including in facilities located in Mombasa city more than 60 kilometers away. In a separate survey carried out in this population, we established that the number of males above 20 years of age was disproportionately lower than females, implying male labour out-migration in this population. It is likely that households with such a family member (financially capable/working) would seek services in urban locations far from home, where the working family member resided. Alternatively, it was possible that households members could travel to seek services in more urban areas in the belief that urban health facilities offered better services and were better resourced than those in rural areas [5,23,24].

We observed that majority of the respondents visited dispensaries when they fell ill. This finding is in concurrence with studies from other LMICs which show that the majority of the poor tend to utilize primary healthcare (PHC) services [5,25], though healthcare resources are concentrated in higher levels (tier 4/district hospital) of the health system which more often serve higher income groups. Kenya spends 50% of her healthcare budget in tier 4 facilities, benefiting the upper two quintiles of the population [26]. In sub-counties of Kaloleni, the majority of the higher level facilities are located in urban areas while the largest proportion of the population is rural. This implies that distance to health facilities could also influence health seeking behaviour in this population as has been shown elsewhere in LMICs [27,28]. We were unable to determine distances to facilities within the scope of this study. While it’s a positive trend that primary care facilities were utilized extensively, the county and sub-county health management should strengthen this level of care to reduce dependence of higher level facilities by e.g. ensuring allocation of resources according to need and demand.

Access and utilization of health services was inequitable even within households. Relatives other than the head of the household and their immediate (nucleus) family were significantly disadvantaged in utilizing health services when they fell ill, although other unmeasured household-level factors could account for a third of the variation in this outcome. This observation was probably influenced by household power dynamics and prevailing social norms e.g. a daughter-in-law may not request her father-in-law for money to take her sick child (or herself) to a hospital. This being a mainly patriarchal community, male heads of the household have the most authority on matters affecting household members including those related to seeking health sevices for their spouses [29]. In this case, household heads may favour their more immediate families in decisions related to seeking healthcare. Related to this, poverty could play an important role in this observation, in which the household head may confer priority to his immediate family members to expend the scarce household resources on healthcare at the expense of other members of the extended family. The local health system management, through the existing community health strategy and partnerships with NGOs working in this community should target these disadvantaged groups with programmatic interventions aimed at improving access to health services for them. The CHWs could be trained to target and place emphasis on these groups during their routine health promotion work in the community.

### Limitations of the study

We did not measure distance to the nearest health facility, and this has been shown to influence access to and utilization of health services. In low income populations however, distance is mainly related to cost of travel (rather than time taken) and is therefore reflected in the reasons given for failure to seek care, although these could not be included in the regression analyses. Our multilevel model (when adjusted for interaction between age and relationship to head of the household) failed to converge on the ‘child’ category of the relation to head of household as none (0) of those who failed to visit health facility when were 'child' of the hhh in the '50+' year age category.

Most of the respondents were male household heads and this could have biased the responses, for instance it is likely that the male respondents may have been unaware of health status of some household members.

Signs and symptoms of the reported ill health episodes were self-reported and as such not classifiable into distinct disease entities. We however grouped these broadly based on the organ system affected and/or on whether they were possibly infectious or non-infectious and were therefore able to identify infectious conditions as the most common causes of morbidity. Also utilization may have varied depending on whether the index illness was a chronic/non-communicable or acute condition but this was difficult to determine given that only signs and symptoms were reported.

## Conclusion

We have generated new information that enhances our understanding of utilization of health services in our study population. This information will enrich the perspective of the local health management to better plan the allocation of scarce healthcare resources to the level of health system that requires them the most, i.e. those with the highest patronage by the population. We have also identified household-level factors that influence utilization of health services during an illness. These are related to relationships within the household and possibly influenced by household power dynamics and economic considerations (poverty related factors) when expending household resources on health services. The local health management together with other stakeholders should target the disadvantaged groups through the health promotion work of CHWs already working in this community. Ultimately, these data shed light on some of the key issues that can be targeted in efforts to improve equity in access and utilization of health services in this population.

## Supporting information

S1 Appendix (DOCX)

S1 Table (DOCX)

S2 Table (DOCX)
