# Supplementary material for: Utilization of health services in a resource-limited rural area in Kenya: Prevalence and associated household-level factors
Source: PLoS One. 2017 Feb 27;12(2):e0172728. doi: 10.1371/journal.pone.0172728 (PMC5328402; doi:10.1371/journal.pone.0172728)
Supplement: S1 Appendix — (DOCX) [file pone.0172728.s001.docx]

# Supporting Information

## S1 Appendix: Questions on history of sickness and utilization of health services in the month preceding the survey

1. Has anyone in your household been sick in the last one month?
2. If YES, how many people in this household have been sick in the last one month?

(Kindly tell me about each person who has been sick in the last one month)

1. List NAMES of all those who were sick in the last one month

*Enumerator: Ask the following for each of those listed:*

1. What SYMPTOMS was (NAME) suffering from?
2. Did (NAME) go to health facility when s/he was sick?
3. If YES, which health facility did (NAME) visit?
4. Was (NAME) referred to that health facility?
5. If YES, who referred (NAME) to that health facility?

*Enumerator: List NAMES of all those that did not visit health facility and ask the following for each:*

If (NAME) did not visit a health facility when they were sick, what was the reason?
