# Supplementary material for: Utilization of health services in a resource-limited rural area in Kenya: Prevalence and associated household-level factors
Source: PLoS One. 2017 Feb 27;12(2):e0172728. doi: 10.1371/journal.pone.0172728 (PMC5328402; doi:10.1371/journal.pone.0172728)
Supplement: S1 Table — (DOCX) [file pone.0172728.s002.docx]

# Supporting Information

## S1 Table: Variance components and inter-class correlation coefficient

| Variance component for household | |  |  |  |
| --- | --- | --- | --- | --- |
| Random-effects Parameters | Estimate (95% CI) | ICC* |  |  |
| household ID (identity) |  |  |  |  |
| sd(_cons) | 0.24 (0.22-0.27) |  |  |  |
| sd(Residual) | 0.34 (0.32-0.37) | 0.34 |  |  |
| *ICC (intra-cluster correletaion coefficient) = sd(cons)^2^/(sd(cons)^2^+sd(resid)^2^ | | | | |
