# Supplementary material for: Utilization of health services in a resource-limited rural area in Kenya: Prevalence and associated household-level factors
Source: PLoS One. 2017 Feb 27;12(2):e0172728. doi: 10.1371/journal.pone.0172728 (PMC5328402; doi:10.1371/journal.pone.0172728)
Supplement: S2 Table — (DOCX) [file pone.0172728.s003.docx]

# Supporting Information

## S2 Table: Correlation between utilizationof health services and potential covariates

| **Covariate** | **Correlation coeff. (p-value)** |
| --- | --- |
| Sex | -0.0323 (0.26) |
| Relation to head of household | -0.1040 (<0.001) |
| Age group | 0.1188 (<0.001) |
| Maternal schooling | -0.0261 (0.37) |
| Wealth quintile | 0.0203 (0.49) |
| Occupation of head of household | -0.017 (0.56) |
